# In Silico Evaluation of Ibuprofen and Two Benzoylpropionic Acid Derivatives with Potential Anti-Inflammatory Activity

**DOI:** 10.3390/molecules24081476

**Published:** 2019-04-15

**Authors:** José A. H. M. Bittencourt, Moysés F. A. Neto, Pedro S. Lacerda, Renata C. V. S. Bittencourt, Rai C. Silva, Cleison C. Lobato, Luciane B. Silva, Franco H. A. Leite, Juliana P. Zuliani, Joaquín M. C. Rosa, Rosivaldo S. Borges, Cleydson B. R. Santos

**Affiliations:** 1 Graduate Program of Pharmaceutical Innovation, Federal University of Amapá, Macapá-AP 68902-280, Brazil; joseadolfo@unifap.br (J.A.H.M.B.); lqfmed@gmail.com (R.S.B.); 2 Laboratory of Modeling and Computational Chemistry, Department of Biological and Health Sciences, Federal University of Amapá, Macapá-AP 68902-280, Brazil; recrisvale@hotmail.com (R.C.V.S.B.); cleison@unifap.br (C.C.L.); luciaanebarros@hotmail.com (L.B.S.); 3 Laboratory of Molecular Modeling, State University of Feira de Santana, Feira de Santana-BA 44036-900, Brazil; moysesfagundes@gmail.com (M.F.A.N.); fhpharm@gmail.com (F.H.A.L.); 4 Laboratory of Bioinformatics and Molecular Modeling, School of Pharmacy, Federal University of Bahia, Barão de Jeremoabo Street, Salvador 40170-115, BA, Brazil; pslacerda@gmail.com; 5 Computational Laboratory of Pharmaceutical Chemistry, University of Sao Paulo, Av. Prof. do Café, s/n - Monte Alegre, Ribeirão Preto, São Paulo 14040-903, Brazil; raics@usp.br; 6 Nucleus of Studies and Selection of Bioactive Molecules, Institute of Health Sciences, Federal University of Pará, Belém-PA 66075-110, Brazil; 7 Laboratory Cellular Immunology Applied to Health, Oswaldo Cruz Foundation, FIOCRUZ Rondônia, Rua da Beira, 7671 BR-364, Porto Velho-RO 78912-000, Brazil; zuliani@fiocruz.br; 8 Department of Pharmaceutical and Organic Chemistry, Faculty of Pharmacy, Institute of Biosanitary Research ibs.GRANADA. University of Granada, 18071 Granada, Spain; jmcampos@ugr.es

**Keywords:** inflammation, molecular docking, molecular dynamics, bioavailability, toxicity

## Abstract

Inflammation is a complex reaction involving cellular and molecular components and an unspecific response to a specific aggression. The use of scientific and technological innovations as a research tool combining multidisciplinary knowledge in informatics, biotechnology, chemistry and biology are essential for optimizing time and reducing costs in the drug design. Thus, the integration of these in silico techniques makes it possible to search for new anti-inflammatory drugs with better pharmacokinetic and toxicological profiles compared to commercially used drugs. This in silico study evaluated the anti-inflammatory potential of two benzoylpropionic acid derivatives (MBPA and DHBPA) using molecular docking and their thermodynamic profiles by molecular dynamics, in addition to predicting oral bioavailability, bioactivity and toxicity. In accordance to our predictions the derivatives proposed here had the potential capacity for COX-2 inhibition in the human and mice enzyme, due to containing similar interactions with the control compound (ibuprofen). Ibuprofen showed toxic predictions of hepatotoxicity (in human, mouse and rat; toxicophoric group 2-arylacetic or 3-arylpropionic acid) and irritation of the gastrointestinal tract (in human, mouse and rat; toxicophoric group alpha-substituted propionic acid or ester) confirming the literature data, as well as the efficiency of the DEREK 10.0.2 program. Moreover, the proposed compounds are predicted to have a good oral bioavailability profile and low toxicity (LD_50_ < 700 mg/kg) and safety when compared to the commercial compound. Therefore, future studies are necessary to confirm the anti-inflammatory potential of these compounds.

## 1. Introduction 

The organism is constantly subject to stimuli and aggressions that can cause infections and tissue damage. Therefore, as a defense mechanism, the biological system developed an adaptive response that seeks to circumvent a tissue aggression, called inflammation. Inflammation is a complex event that involves a perfect and coordinated cascade of cellular and molecular events that aims to remove the harmful stimulus and restore damaged tissue. As a result of these events, the formation of arachidonic acid (AA) occurs from phospholipids of cell membranes under the action of the enzyme called phospholipase A2 (PLA2) [1,2,3,4,5].

Acute inflammation is a short duration process (hours to months), and involves mediators such as prostanoids and nitric oxide (NO) substances that provoke the initial classic signs of the inflammatory process (pain, heat, flushing and edema, with or without loss of function in the tissue of the affected organ) [6,7]. Acute inflammation may be terminated by resolution of all events characteristic of the inflammatory reaction and return of the injured tissue to normality or its replacement by connective tissue [8,9].

If the inflammatory process progresses, there is the development of chronic inflammation, which can last from weeks to years, and is characterized by an infiltrate of mononuclear cells, which include macrophages, lymphocytes and plasma cells. During chronic inflammation, there is usually an active inflammatory process, attempts at tissue repair and tissue destruction, and formation of fibrosis and chronic inflammation is involved in several pathologies such as rheumatoid arthritis and atherosclerosis, among others [10,11,12].

Among the main classes of drugs used in the treatment of inflammatory processes are propionic acid derivatives, among them ibuprofen (IBP). This class of drug is a category of nonselective, nonsteroidal anti-inflammatory drugs (NSAIDs) and they reduce pain (analgesia), body temperature in fever (antipyretics), signs of inflammation (anti-inflammatory activity) and, in mice, slow the development of cancers and have less severe adverse effects than acetylsalicylic acid and indomethacin (gastric discomfort, nausea and vomiting), which are more tolerated by the patient. Despite the therapeutic objectives of non-steroidal anti-inflammatory drugs (NSAIDs) such as rheumatic diseases, heat, erythema, edema and pain, polemics about efficacy and especially toxicity still lead to many discussions [13,14,15,16,17].

Importantly, the analgesic and anti-inflammatory effects of IBP are thought to arise from the inhibition of COX-2 rather than COX-1 [18]. In order to compare the binding mode of IBP to COX-2 versus COX-1, and to reveal a possible mechanism of IBP-mediated substrate selective inhibition, Orlando et al. [19,20] determined the crystal structure of murine (mu) COX-2 in complex with IBP.

The cyclooxygenases (COX-1 and COX-2) catalyze the rate-limiting step in the biosynthesis of prostaglandins, and are the pharmacological targets of non-steroidal anti-inflammatory drugs (NSAIDs) and COX-2 selective inhibitors (coxibs). IBP is one of the most commonly available over-the-counter pharmaceutical in the world. The anti-inflammatory and analgesic properties of IBP are thought to be from the inhibition of COX-2 rather than COX-1 [19,20].

The high financial costs and high time required for the production of drugs are worrying factors for the growth of the pharmaceutical industry, which requires R&D investments for the discovery of new drugs with greater efficiency and selectivity. An expenditure of $800 million to $1.4 billion and about 15–25 years was estimated for the development of a new drug [21,22]. Thus, in order to optimize time and reduce new costs, the use of scientific and technological innovations as a research tool combining multidisciplinary informatics, biotechnology, chemistry and biology knowledge is essential for the pharmaceutical industry of new drugs [23,24,25].

**Figure 1:**
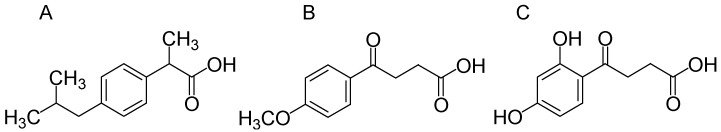
The 2D structures of the ligands used in this study: Ibuprofen (**A**); 4-(4-methoxyphenyl)-4-oxobutanoic acid—MBPA (**B**), and 4-(2,4-dihydroxyphenyl)-4-oxobutanoic acid—DHBPA (**C**).

The aim of this study was to evaluate in silico ibuprofen and two benzoylpropionic acid derivatives (MBPA and DHBPA) with potential anti-inflammatory activity via molecular docking with the COX-2 receptor (PDB codes 4PH9) using the GOLD program [26,27] to evaluate the possible action mechanism of the studied compounds compared with ibuprofen, see Figure 1. In addition, we characterized their thermodynamic profiles by molecular dynamics using the MMPSA routine via GROMACS v4.5.6 [28] and to predict oral bioavailability and bioactivity using the Molinspiration server, see site http://www.molinspiration.com, as well as, an analysis of toxicity through server Protox, see website http://tox.charite.de/tox/, followed by the Derek Nexus program (LHASA LIMITED). The molecular design and conception of bioactive molecules from aryl-acetic and aryl-propionic acids studied here as new derivatives of nonsteroidal anti-inflammatory drugs (NSAIDs) are provided forward (see more details in the materials and methods section).

## 2. Results and Discussion

### 2.1. Molecular Docking Studies

Molecular docking studies were employed to predict the affinity binding of ibuprofen derivatives into a cyclooxygenase isoform COX-1 or COX-2 binding site. Predicting binding geometries and affinities about compound/protein complex is a challenge in structure-based drug design. Stochastic methods (e.g., genetic algorithm) allow for building flexibly different conformers in the active site through the changes of a rotatable bonds angle, and thus, searching for reasonable binding poses of a compound. In addition, the heuristic of the genetic algorithm has an accuracy superior to non-flexible methods [29].

GOLD program uses the genetic algorithm to consider the ligands and active site flexibility and predict satisfactory binding geometries in order to find the most stable conformer [27,28]. Thus, the GOLD program was employed on docking studies. After the search to satisfactory solutions, GOLD can score the conformers affinity in the active site by the definition of the force field and intermolecular forces (e.g., GoldSCORE), improving the potential of interactions to reproduce biological data from a training set (e.g., ChemPLP and ChemSCORE), or build statistical potentials from the contacts frequency of atoms present in the ligand-protein complex (e.g., ASP) [27,28,29].

One way to probe the ability of scoring functions is analyzing the best pose obtained through the program and the conformation of crystallographic ligand [30]. When root-mean-square deviation of atomic positions (RMSD) between them is less than 2 Å, the model achieves satisfactory solutions (conformational search and scoring functions), according to the literature data [31,32,33,34,35,36,37,38,39]. 

All score functions implemented on the GOLD program showed RMSD values less than 2 Å (ASP, RMSD = 0.69; ChemSCORE, RMSD = 1.09; GoldSCORE, RMSD = 0.50; and ChemPLP, RMSD = 0.34). In fact, the RMSD value is not enough to discriminate one score function to employ on docking. In order to circumvent this problem, other parameters were employed to probe the scoring functions ability. Thus, the receiver operating characteristic (ROC) analysis probed the differentiation capacity of active compounds (sensitivity) and similar compounds without biological activity (specificity) on docking by scoring functions.

**Figure 2:**
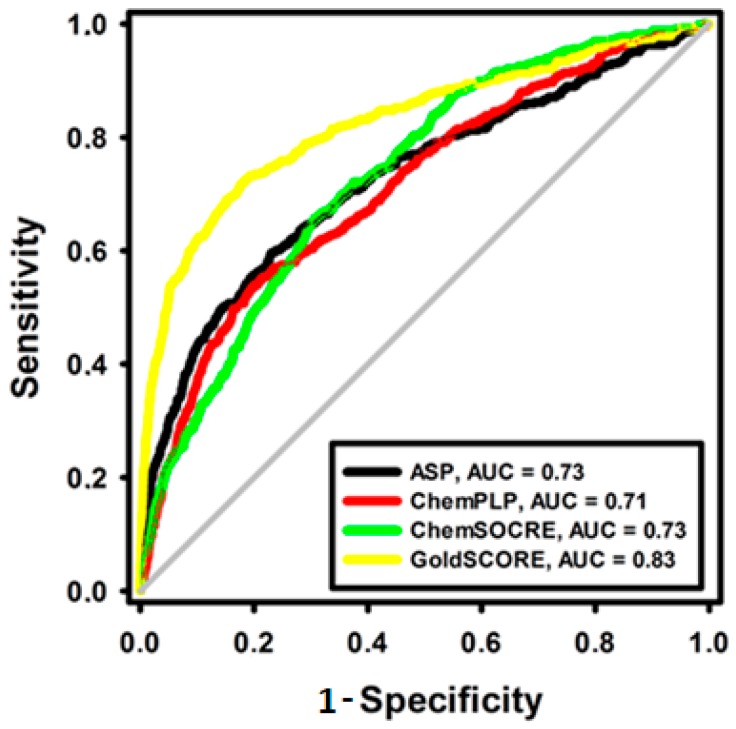
Receiver operating characteristic (ROC) curves of fitness functions implemented in the GOLD program. The diagonal line represents a model that would perform no better than random (AUC = 0.5).

The docking with each GOLD fitness function was performed with a database of active compounds and false-positives (decoys) from the DUD server (426 actives to 13.289 decoys). From the alignment results, the score values of each molecule were used to build the ROC curve, creating a sensitivity (active axis) and specificity (decoys axis) relationship and measuring the area under the curve (AUC), which shows the ability of the fitness function to recognize only actives than decoys when the AUC is equal to 1.0, while an AUC < 0.5 means that the scoring function is worse than a randomized assay, considering functions with AUC ≥ 0.7 with a good discriminatory power [40,41]. All fitness functions implemented on the GOLD program showed an AUC > 0.7 (Figure 2).

Although all score functions had good discriminatory power (AUC > 0.7), the ROC curve is a continuous metric, thus, it is not able to rank active compounds orderly in their initial steps [42]. In addition, this only metric is not able to discriminate one score function, so a Boltzmann enhanced discrimination of the ROC curve (BEDROC) was employed to probe the ability of early and ordered recognition of active compounds. In addition, BEDROC is performed by an exponential function (α), which specifies the early recognition region analyzed, where α = 16.1 is 10% of actives and decoys database. When all active compounds of an analyzed region is ranked and ordered earlier than decoys, BEDROC = 1.0, while a BEDROC < 0.5 means that there is no early recognition by the fitness function [43].

As fitness functions with a BEDROC value > 0.50 are considered better than a random selection, thus, useful for prioritization of active compounds, only GoldSCORE (BEDROC = 0.51) had early and ordered recognition ability, see Table 1.

**Figure 3:**
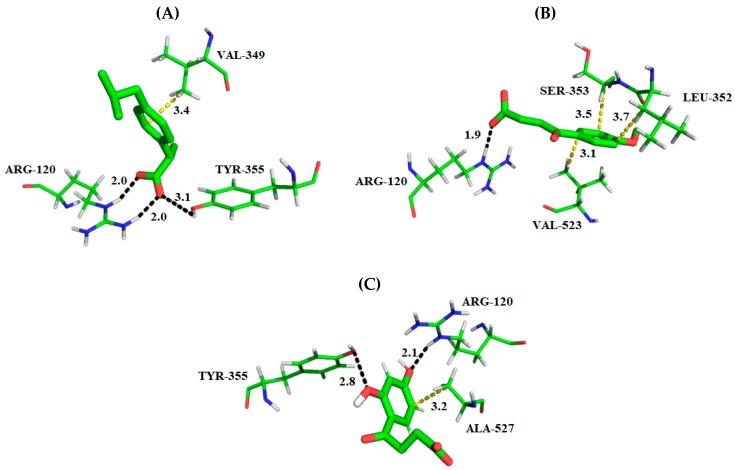
3D Interaction maps (distances Å) of derivative compounds and crystallographic ligand with a COX-2 active binding site; (**A**) experimental binding profile of ibuprofen, (**B**) best ranked pose of MBPA and the (**C**) best ranked pose of DHBPA. The substrate site residues are depicted in lines, whereas compounds are depicted in the stick model. The hydrogen bonds are displayed as black dashed lines and hydrophobic interactions in yellow dashed lines. All distances are measured in angstroms. Stick: Green = carbon, red = oxygen and gray = hydrogen. Lines: Green = carbon, red = oxygen, gray = hydrogen and blue = oxygen. Figure was generated using the PyMOL 2.2.3 program.

Among the fitness functions probed, GoldSCORE is the original fitness function provided with GOLD program, which takes into account factors, such as, H-bonding energy, van der Waals energy, metal interaction and ligand torsion strain [27]. Based on the evaluated parameters, the interaction mode of best conformers (DHBPA and MBPA) with COX-2 is described below, see Figure 3.

The carboxyl group of ibuprofen (PDB ID 4PH9) made a hydrogen bond with the phenol of Tyr355 (donor) and guanidinium group of Arg120 (donor), while Val349 made hydrophobic interactions with the aryl/alkyl group (Figure 3A). Using the docking search parameters selected, we identified a potential binding mode for the synthetic compounds capable of interacting with the COX-2 active site. These interactions are similar to the observed for ibuprofen crystallographic pose around the α-helix located between the amino acid residues Asn87-Thr94, Lys115-Ser122, Val350-Ser354, Met523-Lys533 as well as around the β-sheet located between the amino acid residues Gly355-Phe358, Leu385-Trp388 and Ala517-Gly520. For the compounds studied, it is possible to observe common hydrogen bonds formed with residues Arg120 and Tyr355. There are also hydrophobic interactions with residues Val117, Val350, Trp388, Met523, Val523, Gly527, Ala527, Ser531 and Leu532 according to a study found by Orlando et al. [19].

In comparison with MBPA, the carboxyl group of compound (docking score = 48.79) made a hydrogen bond with the guanidinium group of Arg120 (donor; α-helix located). While the aryl group made hydrophobic interactions with Leu352 and Ser353 (β-sheet located) and Val523 (α-helix located). The hydroxyls of aromatic group of DHBPA (Docking score = 42.04) made a hydrogen bond with the guanidinium group of Arg120 (donor) and phenol group of Tyr355 (acceptor, β-sheet located). Ala527 (α-helix located) made hydrophobic interactions with the phenol group of compounds (Figure 3). The interactions maps of DHBPA and MBPA compounds showed that the presence of hydrogen bond acceptor/donor features of the phenyl group with hydrocarbon carboxylic is important for COX-2 inhibition, due the hydrogen bond with Tyr355 and Arg120. In addition, this is observed on propionic acid derivative drugs, which is the chemical pattern of many COX-2 inhibitors. Although docking studies have proved useful to predict the binding geometries of MBPA and DHBPA compounds, the complete flexibility of the complex was not accounted, because the docking does not even take into consideration the solvation/desolvation effects. In order to overcome such limitations, molecular dynamics simulations were carried out.

### 2.2. Molecular Dynamics Simulations, RMSD and Trajectory Analysis 

**Figure 4:**
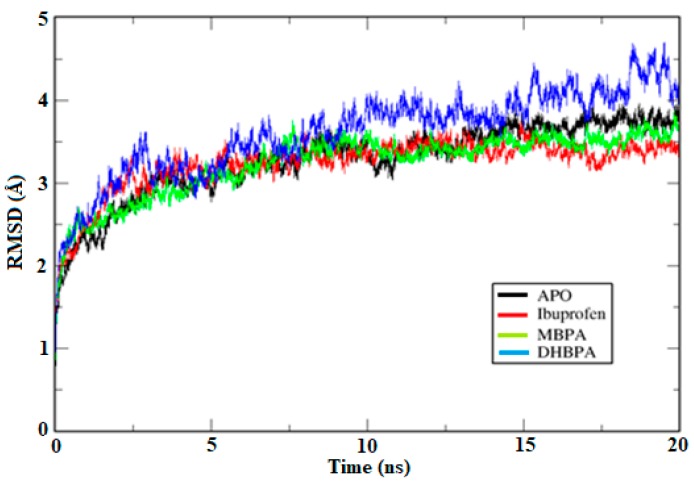
Root-mean-square deviation of atomic positions (RMSD) graph of COX-2 with compounds plotted in the XMGrace program.

In order to evaluate the structural stability of the COX-2 model to perform molecular dynamic (MD) simulations of ligand/protein complexes (ibuprofen/COX-2, DHBPA/COX-2 and MBPA/COX-2), the RMS function implemented on GROMACS was employed to calculate the root-mean-square deviation of atomic positions (RMSD) of the protein, and quantify structural modifications along the simulation (Figure 4).

**Figure 5:**
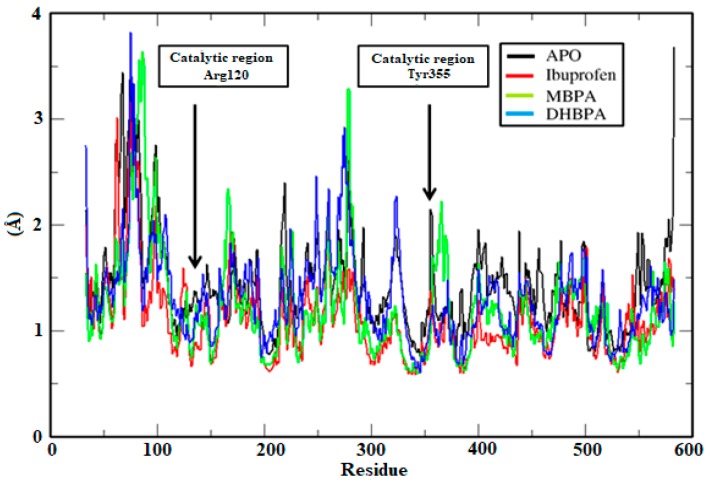
A root-mean-square fluctuation (RMSF) graph of COX-2 with compounds plotted in the XMGrace program.

Comparison of the RMSD values of complexes with the APO structure (RMSD = 3.26 Å) suggests that ibuprofen and MBPA were stable with 10 ns (3.23 Å and 3.23 Å, respectively). Although DHBPA (RMSD = 3.61 Å) showed slight structural divergences after 10 ns, the average by RMSD is highly influenced by flexible regions (e.g., loops) and thus, the complex was not discarded of further analysis. This way, comparison of the root-mean-square fluctuation (RMSF) of the binding site residues (Arg120 and Try355) in APO (RMSF = 1.44 Å) and complex systems suggests that ibuprofen, MBPA and DHBPA (1.43 Å, 1.22 Å and 1.30 Å, respectively) binding reduces residues fluctuation (Figure 5).

**Figure 6:**
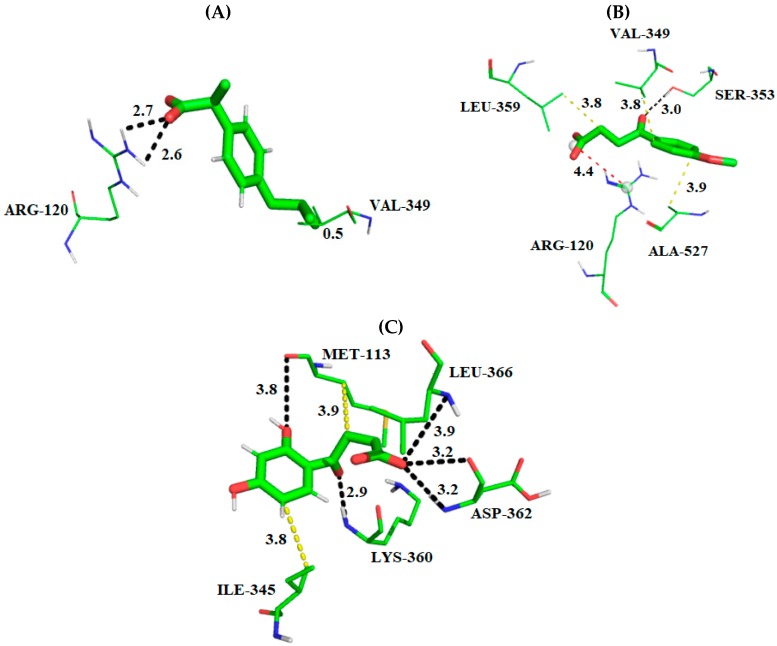
3D interaction maps (distances Å) of all compounds studied with a COX-2 active binding site from 20 ns molecular dynamic simulation; (**A**) binding profile of ibuprofen, (**B**) binding profile of MBPA compound and (**C**) binding profile of DHBPA compound. The substrate site residues are depicted in lines, whereas compounds are depicted in the stick model. The hydrogen bonds are displayed as black dashed line, hydrophobic interactions are displayed as yellow dashed line and the saline bridge as a red dashed line. All distances are measured in angstroms. Stick: Green = carbon, red = oxygen and gray = hydrogen. Lines: Green = carbon, red = oxygen, gray = hydrogen and blue = oxygen. Figure was generated using the PyMOL 2.2.3 program.

Taking the RMSD and RMSF results into consideration, the visual analysis of representative MD structure was carried out to investigate the interaction profile of complexes. Visual analysis of the ibuprofen complex representative MD structure (Figure 6A) showed that the carboxyl group of inhibitor made hydrogen bonds with Arg120 (acceptor; α-helix located), while Val349 (α-helix located) made hydrophobic interactions with the compound. The carboxyl group of MBPA made a hydrogen bond with Ser353 (donor; α-helix located), while the carbonyl group of the compound made a saline bridge with Arg120 (donor; α-helix located). The residues Ala527 and Val349 (α-helix located) and Leu359 (β-sheet located) made hydrophobic interactions with MBPA (Figure 6B). DHBPA compound made a hydrogen bond with Met113 (acceptor), Lys360 (donor), Asp362 (donor) and Leu366 (donor), while Met113 and Ile345 made hydrophobic interactions with the compound (Figure 6C).

Despite that some interactions differ between the best docking poses and complex representative MD structures, this sort results is based in a single structure analysis. Short simulations (10–20 ns) would be enough to investigate the complex stability on the productive phase [44], this way the energy of interactions between COX-2 and ibuprofen, MBPA and DHBPA were investigated.

### 2.3. Binding Free Energy

One of the main challenges in structure-based methods is to be able to predict binding affinities for putative receptor ligand complexes, because the traditional methods (e.g., free energy perturbation and thermodynamic integration) are computationally expensive [45]. 

This way, methods able to use an ensemble of structures at the initial and final states (e.g., MM/PBSA) make these approaches computationally highly efficient. MM/PBSA uses the molecular dynamic trajectories in order to incorporate conformational fluctuations and entropic contributions to the binding free energy estimate and evaluate the relative stabilities of complexes. In addition, this approach is one of the most widely used methods employed in biomolecular complexes studies by decomposing the total binding energy into a series of components [45].

MM/BPSA was employed to predict the free binding energy of the complexes on the last 10 ns of trajectory, which DHBPA and MBPA showed negative values of binding free energy, similarly to the value obtained of crystallographic ligand of COX-2, see Table 2.

Binding contribution is associated with a reduction in the mobility of the active site flaps, where the stability of the complex is expected. The results of components that were favorable to the binding interaction suggest that DHBPA and MBPA were efficient as COX-2 inhibitors, thus signalized by the low values of potential energy (E_MM_) in comparison with ibuprofen (E_MM_ > −34.97 kcal/mol).

Despite the components that are favorable to binding interaction, the contributions of the desolvation of polar and nonpolar groups is unfavorable and made a penalty in binding affinity [46]. DHBPA had the higher value of polar solvation energy (G_polar_ = 16.91 kcal/mol) in comparison with ibuprofen (G_polar_ = 9.52 kcal/mol), while the nonpolar contributions were similar for all compounds. This way, DHBPA was penalized on the binding free energy and had ΔG_Bind_ less negative than ibuprofen value (−24.01 and −28.69 kcal/mol, respectively). On the other hand, the MBPA compound showed the best value of binding free energy (−35.50 kcal/mol), as well as a good fit to the active site.

### 2.4. In Silico Study of Oral Bioavailability, Bioactivity and Toxicity Risk Assessment

The success rate of new candidates selected for the clinical development of drugs in the process of discovery and development is approximately 20% [20,21,22]. This is due mainly to the low rate of absorption, high level of elimination and hepatic clearance that causes a low bioavailability and, consequently, making it difficult to reach the development stage [47].

Molecular descriptors established by the Lipinski rule of benzoylpropionic acid derivatives (Figure 1) are described in Table 3. The compounds MBPA and DHBPA have a molecular weight (MW) of 208.21 and 210.19 Da, respectively. Many of the commercially available drugs have a molecular weight (MW) <500 Da and this facilitates the process of transporting, diffusing and absorbing the drugs when compared to substances with a higher molecular weight (MW > 500) due to increasing the volume of the drug molecule because increasing molecular weight makes the drug absorption process difficult. However, some drugs with a higher molecular weight (MW > 800) than that established by Lipinski’s rule (<500 Da) may show good absorption since they have a good liposolubility profile [48,49].

Another factor that influences the absorption process is the presence of hydrogen bond acceptors (O and N atoms) and hydrogen bond donors (NH and OH) that favor interactions of type dipole-induced or London forces or van der Waals. The presence of large numbers of these groups ends up hampering passage through the cell membrane. In our study the compounds of this study found within the Lipinski limit of HBA ≤ 10 and HBD ≤ 5, respectively, see Table 3.

The octanol/water partition coefficient (Log P) was calculated by the methodology developed by Molinspiration that uses the sum of contributions based on fragments and correlation factors [50,51]. LogP is used as a measure of molecular hydrophobicity influencing solubility and, consequently, drug permeability [50,51]. For the substances analyzed, the log P value ranged from 0.68 to 3.46. So, the limit of Lipinski’s rule was acceptable for all compounds. 

The polar molecular surface area (MPSA) is defined as a sum of polar atomic surfaces (usually oxygen, nitrogen and bound hydrogen) in a molecule that has been shown to be a good descriptor that characterizes drug absorption, including intestinal absorption, bioavailability, Caco-2 permeability and blood-brain barrier (BBB) penetration and the values are shown in Table 3 ranging from 37.30 to 94.83 Å^2^. In addition to this parameter, the molecular volume (MV) is also important for determining the transport characteristics of the molecules, such as intestinal absorption or BBB penetration [52,53,54].

The activity of ibuprofen and benzoylpropionic acid derivatives against the GPCR ligand, ion channel modulator, kinase inhibitor, nuclear receptor ligand, protease inhibitor and enzyme inhibitory activity were studied and summarized in Table 4. The molecules having a bioactivity score more than 0.00 are likely to possess considerable biological activities, according to Roy et al. [55] values −0.50 to 0.00 are expected to be moderately active and if the score is less than −0.50, it is presumed to be inactive. 

Ibuprofen and benzoylpropionic acid derivatives showed a moderate bioactivity score for action for the ion channel modulator. This class of receptor is fundamental for the exchange of charged particles through the cell membranes and are important therapeutic targets in the inflammatory process, since they are involved with the modulation of the inflammatory process [56,57,58].

The evaluated molecules have no potential to act as kinase inhibitors as seen through the activity prediction analysis, since they presented a variation from −0.82 to −0.68. The evaluated molecules have no potential to act as inhibitors of kinase, as seen through activity prediction analysis, since they presented a negative score of bioactivity variation of −0.82 at −0.68. Thus, these new candidates will not be able to act as inhibitors of kinases and will not allow modulation of the signaling process involved in the mechanism of diseases such as asthma, diabetes, cancer, cardiovascular diseases and others [32,59].

Nuclear receptors are a class of intracellular transcription factors activated by ligands such as glucocorticoid and steroidal hormones and exert actions such as cell proliferation, development, metabolism and reproduction, and may represent a therapeutic alternative for the treatment of neurodegeneratives pathologies, such as Alzheimer’s disease. However, MBPA and DHBPA showed a moderate bioactivity score for the nuclear receptor ligand, in the range of −0.34 to −0.04. Thus, these molecules need to be better analyzed to verify their activity as a possible drug candidate that acts on nuclear receptors [60,61].

All compounds, ibuprofen, MBPA and DHBPA, presented a bioactivity score for enzymatic inhibition since the score presented values higher than 0.00, according to Table 4. The inflammation process involves a complex, dynamic, well-orchestrated series of events depending on how the various biologic mechanisms are linked [1,2,3,4,5].

Thus, an important mechanism for the beginning of this event is due to the role of cyclooxygenases, whereby all compounds have been shown the capacity to inhibit enzymes, we may assume that one of the mechanisms involved in the inflammatory response is associated with the inhibition of cyclooxygenase and this is important because the use of compounds that present the ability to inhibit enzymes can be used as a model for the development of new drugs with anti-inflammatory activity reference [1,2,3,4,5].

Table 5 shows clearly that LD_50_ prediction value was 299 mg/kg for ibuprofen and 700 mg/kg for MBPA and DHPA and was defined according to the globally harmonized system of labeling of chemicals (GHS) [62]. The compounds of the present study have an advantage over the commercial compound since ibuprofen has an LD_50_ of 299 mg/kg while the compounds evaluated showed LD_50_ of 700 mg/kg. This discloses that such compounds may have greater safety in use, according to the data shown in Table 5.

The compounds MBPA and DHBPA cause toxicity at a higher concentration than the commercial compound. Thus, in future experimental studies, ibuprofen should be administered with greater caution to avoid any loss of animal due to toxicity which, in turn, may affect the analysis of the experiment.

Table 6 shows the results of toxicity predictions by the identification of toxicophore groups of the ibuprofen, MBPA and DHPA. The deductive estimation of risk from existing knowledge (DEREK) 10.0.2 program [35,39,63] is a specialist system that focuses its attention on the toxic action of chemical compounds. The system performs this analysis based on implemented rules and depicts the relationship between a structural feature and a toxicophore group present in the compounds as possible inducers of certain types of toxicity [63].

Anti-inflammatory drugs derived from 2-arylacetic and 3-arylpropionic acids, such as ibuprofen, may cause hepatotoxicity and irritation of the gastric mucosa. The hepatotoxicity is due to an idiosyncratic metabolic and/or immune reaction not associated with a dose and it was observed that mice treated with benoxaprofen developed this problem [64]. In studies carried out by Geneve et al. [65] high doses of pirprofen and ibuprofen can compromise the beta-oxidation process of fatty acids and cause hepatic microvesicular steatosis and this can result in elevations of serum transaminases to severe hepatocellular and/or cholestatic lesions, which due to its seriousness may lead to a clinical process of fulminant hepatic failure [66]. Moreover, we can find jaundice, centrilobular necrosis, microvesicular steatosis, fibrosis and symptoms suggestive of a hypersensitivity syndrome are clinical findings associated with the use of these agents [65,66].

Derivation of modified protein adducts is thought to be essential to the hepatotoxicity induced by these agents. Two alternative metabolic pathways may play a causative role: Hepatic acyl glucuronidation catalyzed by the uridine diphosphoglucurosyl transferase (UGT) system, and acyl coenzyme A (acyl-CoA) formation. It is well established that acyl glucuronides are reactive electrophiles which can undergo covalent u with plasma or tissue proteins via a transacylation or glycation mechanism [67,68]. In the former, protein adducts are formed by nucleophilic displacement of the glucuronic acid moiety. In the latter, intramolecular migration of the acyl residue allows opening of the glucuronic acid ring to create an aldehyde intermediate susceptible to nucleophilic attacks [67,68,69].

Furthermore, NSAIDs, including propionic acid derivatives, are associated with irritation of the gastric mucosa. This is due to the mechanism of action of these compounds, which inhibits the enzyme cyclooxygenase that causes the rupture of the physical-chemical barrier that protects the gastric mucosa and, consequently, causes gastric irritation [70,71].

Already MBPA showed an alert of skin sensitization for phenols. In various skin sensitization assays including the guinea pig maximization test and the human maximization test this effect was demonstrated [72,73]. Phenol itself is corrosive but not a sensitizer [74,75].

The presence of a grouping, which may give rise during the metabolism to the phenol grouping, caused the alertness for skin irritation of the MBPA. This sensitivity is thought to arise from the formation of a radical phenolic which subsequently reacts with skin proteins, primarily at positions ortho to the phenol group [76].

## 3. Materials and Methods

### 3.1. Compounds Studied

**Figure 7:**
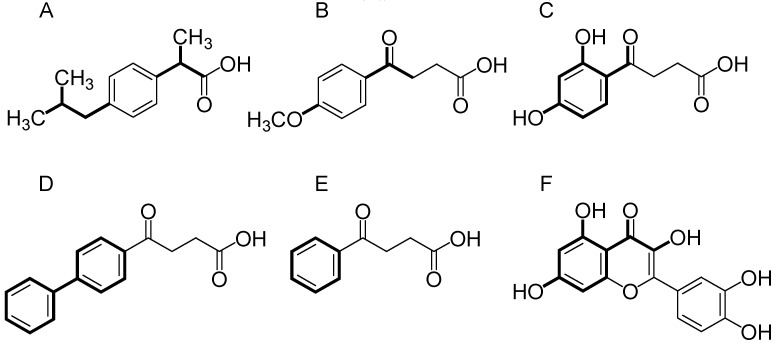
Aryl propionic and aryl butyric acid derivatives and modifications strategies. (**A**) Ibuprofen. (**B**) MBPA. (**C**) DHBPA. (**D**) Fenbufen (**E**) Fenbufen derivate. (**F**) Chemical skeleton of a flavonoid. Bold line - key groups of molecular modification strategies

Ibuprofen or 2-(4-isobutylphenyl) propionic acid was developed in the early 1960s, designed to be a safer drug when compared to acetylsalicylic acid [77,78]. Compounds MBPA (Figure 7B) and DHBPA (Figure 7C) were proposed, see the theoretical synthetic route in Supplementary Material. In our proposal, the high nonpolar groups on ibuprofen (Figure 7A) isobutyl and methyl were changed by methoxy (Figure 7B) and ketone (Figure 7C) moieties. In addition, the propionic acid was modified to 4-oxo-butyric acid. Fenbufen (Figure 7D) was developed as NSAID [79] and this scaffold was placed. However, in the 2010s it was withdrawn from markets in the developed world due to liver toxicity [80]. Experimental studies provide additional evidences that fenbufen and its metabolites could be involved in mitochondrial toxicity through inhibition of ATP synthesis [81]. In addition, the steric and lipophilic biphenyl (Figure 7D) moiety was changed to phenyl (Figure 7E) and evaluated for us. Despite your potent anti-inflammatory activity, the toxicity was also present [82]. Therefore, our strategy involves the application of functional safer derivatives related to natural products such as flavonoids (Figure 7F) (see Figure 7). Furthermore, methoxy (Figure 7B) and hydroxyl (Figure 7C) groups increase the polarity and are a new possible group for chemical interactions on the active site of COX. In fact, knowing the liposolubility profile through the in silico predictions makes the understanding of the compound absorption process possible, since it allows us to evaluate the interaction of these compounds with the cellular membranes, since this type of interaction is fundamental for the bioavailability process of the drug.

### 3.2. Molecular Modeling Studies

#### 3.2.1. Molecular Docking

Docking studies were employed in order to search for reasonable binding poses of DHBPA and MBPA compounds with COX-2 receptor using the GOLD program [26,27]. The chemical structure of compounds was converted to 3D using the CONCORD module and energetically minimized through the conjugated-gradient protocol (convergence criterion = 0.001 kcal/mol; maximum iteration = 50,000), using Tripos force field [79], Gasteiger-Huckel charges [83] and an implicit solvent environment (Dielectric constant = 80.0) as available on SYBYL^®^-X 2.0 platform (Tripos Associates Inc., St. Louis, MO, USA) [84].

The X-ray crystallographic structure of COX-2 in complex with ibuprofen employed in study was from Protein Data Bank (https://www.rcsb.org/structure/4ph9; PDB ID 4PH9) and has 1.81 Å of resolution [19]. All water and ions were discarded, and then hydrogen atoms were added in standard geometry using the Biopolymer module from SYBYL-X 2.0 platform [19]. Next, the protonation state of residues were manually checked using the H++ server (Virginia Tech, Blacksburg, VA, USA) [85] with pH = 7.5 [19].

The conformational search and scoring evaluation were performed employing the piecewise linear potential (ChemPLP), GoldSore, ChemScore and Astex statistical potential (ASP) scoring functions as available on the GOLD program (5.4.0, The Cambridge Crystallographic Data Centre, Cambridge, United Kingdom) using the default parameters [26,27]. The ability to generate satisfactory solutions of scoring functions was probed according to the root mean square deviation value (RMSD < 2 Å). Next, the area under the curve (AUC) prediction of the receiver operating characteristic (ROC) was used to measure the capacity of each function to score active and false-positives (decoys) through AUC > 0.7 using the SigmaPlot^®^ v. 12.0 program (Systat Software Inc, Chicago, IL, USA) [86]. Then, the Boltzmann enhanced discrimination of ROC (BEDROC > 0.5) was employed to measure the ability of each function to recognize actives compounds in their initial steps using the ROCKER server (Universitu of Turko, Turku, Finland) [87].

The best pose of the DHBPA and MBPA compound obtained with the best scoring function available on GOLD were employed in molecular dynamics studies. 

#### 3.2.2. Molecular Dynamics (MD)

GROMACS 4.5.6 version package (University of Groningen, Groningen, Groningen Province, The Netherlands) [28] as used to perform molecular dynamics simulations of the apo form, ibuprofen/COX-2 (crystallographic ligand), DHBPA/COX-2 and MBPA/COX-2 complexes, which were obtained from the previous docking step. The topology of each ligand was generated using the PRODRG 2.5 server (GlycoBioChem Ltd., Dundee, Scotland) [88] and used to build the complexes. GROMOS96 force field (53a6) [89] was employed for all simulation. The water molecules (extended simple point charge (SPC/E) model) [90] were inserted into a cubic box at a 1.4 nm distance from the protein surface. Some water molecules were replaced by positive ions (Na^+^) to neutralize the system that were randomly distributed inside the box.

A three-step procedure (5000 steps each) of energy minimization was employed to prepare the system to production molecular dynamics. First, a steepest-descent algorithm was applied, restraining harmonically the protein non-hydrogen atoms to their initial positions; followed by a second steepest descent minimization with all atoms unrestrained. Subsequently, a conjugated gradient algorithm was applied to the entire systems for further energy minimization. 

The bonds involving hydrogen atoms were constrained using LINCS and SETTLE algorithms for protein/ligands and water molecules, respectively. Periodic boundary conditions (PBC) were applied to Coulomb and van der Waals interactions. The long-range interactions were treated using the particle-mesh-Ewald (PME) electrostatic method [91].

A 1000 ps MD equilibration was performed with the protein non-hydrogen atoms position restrained. In this step, a random Boltzmann distribution was used to generate the initial velocities for each simulation. Then, each simulation of complexes performed 20,000 ps in constant temperature (303.15 K) and pressure conditions (1 atm).

The average structure was selected by a clustering algorithm method [28] implemented in GROMACS 4.5.6, with a cut-off of 0.17 nm during the productive phase.

#### 3.2.3. Molecular Dynamics Trajectory Analysis

Root-mean-square deviation of atomic positions (RMSD) was employed to evaluate the structural stability of the APO form and complexes (ibuprofen/COX-2, DHBPA/COX-2 and MBPA/COX-2) by a RMS function implemented in GROMACS 4.5.6 [28]. Next, a root-mean-square fluctuation (RMSF) was employed to evaluate the residue fluctuation of the APO form and complexes (ibuprofen/COX-2, DHBPA/COX-2 and MBPA/COX-2) by the RMS function implemented in GROMACS 4.5.6 [28]. RMSD and RMSF graph were plotted in XMGrace program (WIS Plasma Laboratory, Rehovot, Israel) [91]. Then, the stability of hydrogen bonding interactions was evaluated by the HBOND function implemented in GROMACS 4.5.6 [28] and hbmap2grace.07 (Laboratory for Molecular Modeling and Dynamics, Rio de Janeiro, Brazil) [92].

#### 3.2.4. Binding Free Energy Calculations

The molecular mechanics Poisson−Boltzmann surface area (MM/PBSA) method implemented in the g_mmpbsa tool [45] was employed to quantify the binding free energy of ibuprofen/COX-2 (crystallographic ligand), DHBPA/COX-2 and MBPA/COX-2 complexes in a three-steps procedure to 20 snapshots extracted every 0.5 ns from the production trajectories (10 to 20 ns).

First, the vacuum potential energy (E_MM_) was measured by electrostatic (E_elec_) and van der Waals (E_vdW_) interactions using Coulomb and Lennard-Jones (LJ) potential functions (Equation (1)); Subsequently, the polar solvation energy (G_polar_) of complexes was quantified in a grid box (cfac = 2 and fadd = 20) with 0.150 M NaCl solvent (radii_Na_ = 0.95 Å; radii_Cl_ = 1.81 Å) and dielectric constant = 80 by Debye-Hückel approximation; then, the nonpolar solvation energy (G_nonpolar_) was calculated using a solvent accessible surface (SASA) model with a default surface tension of solvent (gamma = 0.02267 kJ mol ^−1^ Å^−2^) [45,93,94,95].

Standard output includes the binding energy terms of the three components and the binding free energy is calculated following Equations (1) and (2):

E_MM_ = E_vdW_ + E_elec_ (1)

ΔG_Bind_ = E_MM_ − (G_polar_ − G_nonpolar_) (2)

#### 3.2.5. In Silico Study of Oral Bioavailability, Bioactivity and Toxicity Risk Assessment 

The compounds used this study were: Ibuprofen, MBPA and DHPA, molecular descriptors and drug likeliness (oral bioavailability) properties of the compounds were analyzed via the Molinspiration server, see site http://www.molinspiration.com, based on the Lipinski Rules of five [48,49].

The rule states that most “druglike” compounds have a molecular weight (MW) ≤500 Da, number of hydrogen bond acceptors (HBA) ≤10, number of hydrogen bond donors (HBD) ≤5 and octanol/water partition coefficient (log P) ≤5. Compounds violating more than one of these rules can have problems with oral bioavailability, Molinspiration supports the calculation of important molecular properties such as (MW, LogP, polar surface area, number of hydrogen bond donors and acceptors), as well as, prediction of the bioactivity score for the most important drug targets (GPCR ligands, kinase inhibitors, ion channel modulators, enzymes and nuclear receptors were predicted in this study) [48,49,54].

To identify any undesirable toxic properties of ibuprofen and benzoylpropionic acid derivatives, the toxicity prediction server (Protox), see site http://tox.charite.de/tox/, was used in this study [96,97]. The prediction was based on the functional group similarity for the query compounds with the in vitro and in vivo validated compounds present in this database. The toxic properties such as toxicity class, toxic fragment generation, LD_50_ values in mg/kg, toxicity targets, drug-relevant properties [c Log P, Log S (Solubility)], molecular weight and overall drug-score were calculated [96,97]. This approach was based on studies by Roy et al. [54]. 

The toxicity profile was also evaluated using the DEREK 10.0.2 program (Lhasa Limited, Leeds, United Kingdom). We have considered alerts of toxicity involving the human species and also classified it as plausible in mammals. It is considered that in addition to toxicity, DEREK can identify aspects related to carcinogenicity, mutagenicity, skin sensitization, irritation, teratogenicity and neurotoxicity [38,39,63].

## 4. Conclusions

According to the in silico predictions, the proposed derivatives have the potential of COX-2 inhibition in human and mice enzymes due to containing similar interactions as observed in the control compound (ibuprofen). Ibuprofen showed toxic predictions of hepatotoxicity (in human, mouse and rat; toxicophoric group 2-arylacetic or 3-arylpropionic acid) and irritation of the gastrointestinal tract (in human, mouse and rat; toxicophoric group alpha-substituted propionic acid or ester) confirming the literature data, as well as the efficiency of the DEREK 10.0.2 program. Moreover, the proposed compounds are predicted to have a good oral bioavailability profile and low toxicity (LD_50_ < 700 mg/kg) and safety when compared to the commercial compound. Therefore, future studies (in vitro or in vivo) are necessary to confirm the anti-inflammatory potential of these molecules.

## Figures and Tables

**Table 1:** Boltzman enhanced discrimination of the ROC curve (BEDROC) values of fitness functions implemented in the GOLD program.

Fitness Function	BEDROC (α = 16.1)
ASP	0.37
ChemSCORE	0.29
ChemPLP	0.43
GoldSCORE	0.54

**Table 2:** Binding free energy and components, calculated using MM/PBSA.

Ligand	E_vdW_ (Kcal/mol)	E_elec_ (Kcal/mol)	E_MM_ (Kcal/mol)	G_polar_ (Kcal/mol)	G_nonpolar_ (Kcal/mol)	ΔG_Bind_ (Kcal/mol)
Ibuprofen	−35.07	0.10	−34.97	9.52	−3.24	−28.69
MBPA	−33.30	−4.41	−37.41	5.40	−3.21	−35.52
DHBPA	−34.25	−3.43	−37.68	16.91	−3.23	−24.01

E_vdW_ = van der Waals energy; E_elec_ = Electrostatic energy; E_MM_ = Vacuum potential energy; G_polar_ = Polar solvation energy; G_nonpolar_ = Nonpolar solvation energy; ΔG_Bind_ = Binding free energy.

**Table 3:** Oral bioavailability properties of ibuprofen and benzoylpropionic acid derivatives.

Name	MW ^a^ (<500 Da)	Molecular Formula	HBA ^b^ (≤10)	HBD ^c^ (≤5)	Log P ^d^ (≤5)	MPSA ^e^ (Å^2^)	MV ^f^ (Å^3^)	NRB ^g^
Ibuprofen	206.28	C_13_H_18_O_2_	2	1	3.46	37.30	211.19	4
MBPA	208.21	C_11_H_12_O_4_	4	1	1.30	63.60	189.18	5
DHBPA	210.19	C_10_H_10_O_5_	5	3	0.68	94.83	179.67	4

^a^ Molecular weight, ^b^ Hydrogen bond acceptor, ^c^ Hydrogen bond donor, ^d^ Logarithm of the partition between of n-octanol and water phases, ^e^ MPSA, ^f^ MV and ^g^ NRB (number of rotatable bonds) were obtained.

**Table 4:** Bioactivity of the ibuprofen and benzoylpropionic acid derivatives ^a^.

Name	GPCR	Ion Channel Modulator	Kinase Inhibitor	Nuclear Receptor Ligand	Protease Inhibitor	Enzyme Inhibitor
Ibuprofen	−0.17	−0.01	−0.72	0.05	−0.21	0.12
MBPA	−0.35	−0.22	−0.82	−0.34	−0.53	0.00
DHBPA	−0.19	−0.09	−0.68	−0.04	−0.43	0.20

^a^ Score Values ≥0.00 = considerable biological activities; Score Values −0.50 to 0.00 = moderately active; score values ≤−0.50 = considerable inactive.

**Table 5:** Toxicity prediction of the ibuprofen and benzoylpropionic acid derivatives.

N^o^	Name	LD_50_ Toxic ^a^	Toxicity Class ^b^
1	Ibuprofen	299 mg/kg	III
2	MBPA	700 mg/kg	IV
3	DHBPA	700 mg/kg	IV

^a^ Values in mg/kg body weight, ^b^ Class III: toxic if swallowed (50 < LD_50_ ≤ 300); Class IV: harmful if swallowed (300 < LD_50_ ≤ 2000) [62].

**Table 6:** Toxicity prediction by the identification of toxicophore groups of ibuprofen, MBPA and DHPA.

Compounds	Toxicity Prediction Alert (Lhasa Prediction)	Toxicophoric Group	Toxicity Alert	Toxicity Prediction (Custom Prediction)
Ibuprofen	Hepatotoxicity in human, mouse and rat	2-arylacetic or 3-arylpropionic acid	PLAUSIBLE	Nothing to declare
	Irritation of the gastrointestinal tract in human, mouse and rat	alpha-substituted propionic acid or ester		
MBPA	Skin sensitization in human, mouse and rat	Substituted phenol or precursor	PLAUSIBLE	Nothing to declare
DHBPA	Thyroid toxicity in human, mouse and rat	Resorcinol or 3-aminophenol	PROBABLE	Nothing to declare
	Skin sensitization in human, mouse and rat	Resorcinol or 3-aminophenol	PLAUSIBLE	

## Supplementary Materials

Theoretical synthetic route in supplementary material are available online.
