# Supplementary material for: In Silico Evaluation of Ibuprofen and Two Benzoylpropionic Acid Derivatives with Potential Anti-Inflammatory Activity
Source: Molecules. 2019 Apr 15;24(8):1476. doi: 10.3390/molecules24081476 (PMC6515000; doi:10.3390/molecules24081476)
Supplement: Supplementary file 1 [file molecules-24-01476-s001.pdf]

molecules

MDPI

Article

In Silico Evaluation of Ibuprofen and Two Benzoylpropionic Acid Derivatives with Potential Anti-Inflammatory Activity

José A. H. M. Bittencourt [1,2], Moysés F. A. Neto [3], Pedro S. Lacerda [4], Renata C. V. S. Bittencourt [2], Rai C. Silva [5], Cleison C. Lobato [2,6], Luciane B. Silva [2], Franco H. A. Leite [3], Juliana P. Zuliani[7], Joaquín M. C. Rosa[8], Rosivaldo S. Borges [1,2,6] and Cleydson B. R. Santos [1,2,6,*]

1 Graduate Program of Pharmaceutical Innovation, Federal University of Amapá, Macapá-AP 68902-280, Brazil; joseadolfo@unifap.br (J.A.H.M.B.);
2 Laboratory of Modeling and Computational Chemistry, Department of Biological and Health Sciences, Federal University of Amapá, Macapá-AP 68902-280, Brazil; recrisvale@hotmail.com (R.C.V.S.B.); cleison@unifap.br (C.C.L); luciaanebarros@hotmail.com (L.B.S);
3 Laboratory of Molecular Modeling, State University of Feira de Santana, Feira de Santana-BA 44036-900, Brazil; moysesfagundes@gmail.com (M.F.A.N.); fhpharm@gmail.com (F.H.A.L.);
4 Laboratory of Bioinformatics and Molecular Modeling, School of Pharmacy, Federal University of Bahia, Barão de Jeremoabo Street, 40170-115 Salvador, BA, Brazil; pslacerda@gmail.com (P.S.L.);
5 Computational Laboratory of Pharmaceutical Chemistry, University of Sao Paulo, Av. Prof. do Café, s/n - Monte Alegre, Ribeirão Preto, São Paulo 14040-903, Brazil; raics@usp.br (R.C.S);
6 Nucleus of Studies and Selection of Bioactive Molecules, Institute of Health Sciences, Federal University of Pará, Belém-PA 66075-110, Brazil; lqfmed@gmail.com (R.S.B.);
7 Laboratory Cellular Immunology Applied to Health, Oswaldo Cruz Foundation, FIOCRUZ Rondônia, Rua da Beira, 7671 BR-364, Porto Velho-RO 78912-000, Brazil; zuliani@fiocruz.br (J.P.Z);
8 Department of Pharmaceutical and Organic Chemistry, Faculty of Pharmacy, Institute of Biosanitary Research ibs.GRANADA. University of Granada, 18071, Granada, Spain; jmcampos@ugr.es (J.M.C. R.).
* Correspondence: breno@unifap.br; Tel.: +55-096-4009-2699

SUPPLEMENTARY MATERIAL

Figure S1. Theoretical synthetic route for the preparation of compound 4-(4-methoxyphenyl)-4-oxobutanoic acid (B).

Figure S2. Theoretical synthetic route for the preparation of compound 2,4-dihydroxyphenyl)-4-oxobutanoic acid (C). DMF (*N*, *N*-dimethylformamide), TIPS-Cl (triisopropylsilyl chloride), Imidazole (i) succinic anhydride and aluminum chloride and DCM (dichloromethane), (ii) TBAF (tetra-*n*-butylammonium fluoride) and THF (tetrahydrofuran).

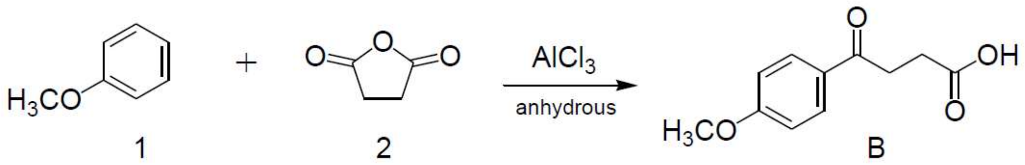

Figure S1. Theoretical synthetic route for the preparation of compound 4-(4-methoxyphenyl)-4-oxobutanoic acid (B).

The 4-(4-methoxyphenyl)-4-oxobutanoic acid (B) was synthesized by anisole with succinic anhydride in the presence of anhydrous aluminum chloride under Friedel-Crafts acylation reaction conditions. The synthesis of 4-(4-methoxyphenyl)-4-oxobutanoic acid has been recently reported by Soliman et al. (2018) [1] is a one-step process and the synthetic strategy known to synthesize efficiently phenyloxobutanoic acids from aromatic hydrocarbon, anisole, in a 1 step process: acylation of anisole with succininc anhydride catalyzed by anhydrous aluminum chloride under conditions of under Friedel-Crafts acylation reaction.

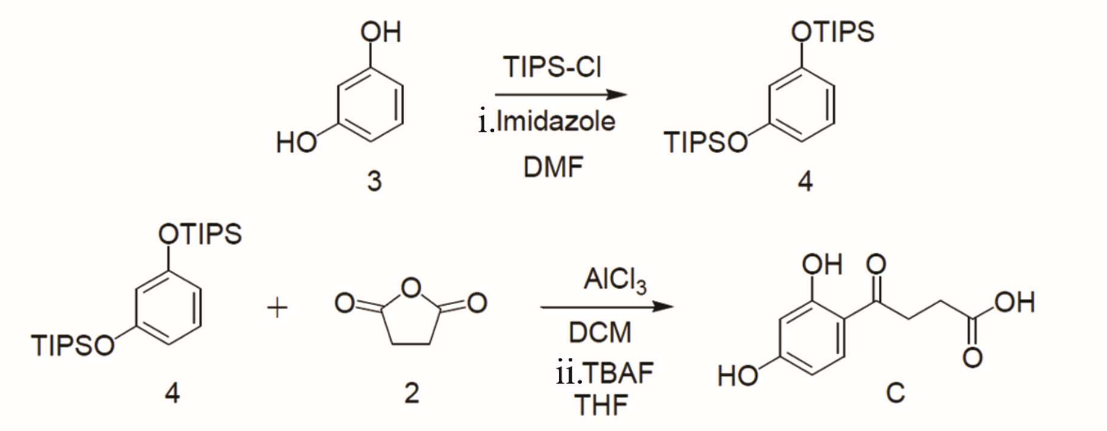

Figure 2. Theoretical synthetic route for the preparation of compound 2,4-dihydroxyphenyl)-4-oxobutanoic acid (C). DMF (*N*, *N*-dimethylformamide), TIPS-Cl (triisopropylsilyl chloride), Imidazole (i) succinic anhydride and aluminum chloride and DCM (dichloromethane), (ii) TBAF (tetra-n-butylammonium fluoride) and THF (tetrahydrofuran).

The synthesis of 2,4-dihydroxyphenyl)-4-oxobutanoic acid (C) has been reported by Liu et al. (2014) [2]. The theoretical synthetic route (See Figure S2) here proposed consists of two steps, in analogy to two variants with carboxylic acid functionalities and anhydrous aluminum chloride in the presence of TBAF (tetra-*n*-butylammonium fluoride), DCM (dichloromethane) and THF (tetrahydrofuran).

In the first step it consists resorcinol e *N*, *N*-dimethylformamide under temperature of conditions that depend on the reaction medium, subsequently, added triisopropylsilyl chloride and imidazole at room temperature for 24 hours. Then, in the second step, in 1,3-*bis*((triisopropylsilyl)oxy)benzene and anhydrous dichloromethane under a temperature of conditions that depend on the reaction medium. Subsequently, succinic anhydride and aluminum chloride are added. The oil formed is dissolved in tetrahydrofuran and will react with tetra-*n*-butylammonium fluoride.

References

1. Soliman, M.H.; Hussein, B.H.; Abdel-Moati, N.; El-Tamany, E.S.H. 3 (2*H*)-Furanones promising candidates for synthesis of new fluorescent organic probes. *Indian. J. Chem.* **2018**, 567–575.
2. Liu, D.S.; Nivón, L.G.; Richter, F.; Goldman, P.J.; Deerinck, T.J.; Yao, J.Z.; Drennan, C.L. Computational design of a red fluorophore ligase for site-specific protein labeling in living cells. *Proc. Natl. Acad. Sci. USA* **2014**, *111*, E4551–E4559, doi:10.1073/pnas.1404736111
